# A Comorbidity Model of Myocardial Ischemia/Reperfusion Injury and Hypercholesterolemia in Rat Cardiac Myocyte Cultures

**DOI:** 10.3389/fphys.2019.01564

**Published:** 2020-01-09

**Authors:** András Makkos, Ágnes Szántai, János Pálóczi, Judit Pipis, Bernadett Kiss, Paola Poggi, Péter Ferdinandy, Alexandros Chatgilialoglu, Anikó Görbe

**Affiliations:** ^1^Cardiometabolic Research Group, Department of Pharmacology and Pharmacotherapy, Semmelweis University, Budapest, Hungary; ^2^Department of Biochemistry, University of Szeged, Szeged, Hungary; ^3^Pharmahungary Group, Szeged, Hungary; ^4^Remembrane S.r.L., Imola, Italy

**Keywords:** cardiac myocytes, ischemia/reperfusion injury (I/R injury), hypercholesterolemia (HC), cell culture, hypercholesterolemia and hyperglycemia

## Abstract

**Introduction:**

The use of comorbidity models is crucial in cardioprotective drug development. Hypercholesterolemia causes endothelial and myocardial dysfunction, as well as aggravates ischemia/reperfusion (I/R)-induced myocardial injury. Endogenous cardioprotective mechanisms against I/R are impaired in hyperlipidemic and hyperglycemic *in vivo* animal models. Therefore, our aim was to develop a medium throughput comorbidity cell-based test system of myocardial I/R injury, hypercholesterolemia and hyperglycemia that mimics comorbidity conditions.

**Methods:**

Cardiac myocytes isolated from neonatal or adult rat hearts were cultured in control or in three different hypercholesterolemic media with increasing cholesterol content (hiChol) or hiChol + hyperglycemic medium, respectively. Each group was then subjected to simulated ischemia/reperfusion (SI/R) or corresponding normoxic condition, respectively. Cholesterol uptake was tested by Filipin staining in neonatal cardiac myocytes. Cell viability, total cell count and oxidative stress, i.e., total reactive oxygen species (ROS) and superoxide level were measured by fluorescent assays.

**Results:**

Neonatal cardiac myocytes took up cholesterol from the different hiChol media at a concentration-dependent manner. In normoxia, viability of hiChol neonatal cardiac myocytes was not significantly changed, however, superoxide levels were increased as compared to vehicle. After SI/R, the viability of hiChol neonatal cardiac myocytes was decreased and total ROS level was increased as compared to vehicle. HiChol combined with hyperglycemia further aggravated cell death and oxidative stress in normoxic as well as in SI/R conditions. Viability of hiChol adult cardiac myocytes was significantly decreased and superoxide level was increased in normoxia and these changes were further aggravated by SI/R. HiChol combined with hyperglycemia further aggravated cell death, however level of oxidative stress increased only in normoxic condition.

**Conclusion:**

HiChol rat cardiac myocytes showed reduction of cell viability and increased oxidative stress, which were further aggravated by SI/R and with additional hyperglycemia. This is the first demonstration that the combination of the current hypercholesterolemic medium and SI/R in cardiac myocytes mimics the cardiac pathology of the comorbid heart with I/R and hypercholesterolemia.

## Introduction

Ischemic heart disease is still the leading cause of death worldwide; therefore, there is an unmet clinical need for the development of efficient cardioprotective therapies. In the last few decades, a wide variety of cardioprotective interventions and pharmacological treatments were found effective in experimental animal models and in cell cultures. However, their clinical translation has been largely disappointing (Hausenloy et al., 2017). One of the major problem is that the *in vitro* preclinical testing of drug candidates apply cell lines and *in vivo*, *ex vivo* testing apply young, healthy animals, thus neglecting the presence of cardiovascular risk factors and comorbidities.

Ischemic heart disease is typically associated with metabolic diseases such as diabetes, obesity, hyperlipidemia and hypercholesterolemia, which predispose the subject to atherosclerosis and the development of coronary artery diseases (CADs) (Benjamin et al., 2017). Hypercholesterolemia is widely accepted as a principal risk factor for CAD (Ferdinandy et al., 2014) and can increase the myocardial damage due to ischemia/reperfusion injury and interfere with responses to cardioprotective interventions (Andreadou et al., 2017). Most of the preclinical studies have shown that hyperlipidemia (but not atherosclerosis) leads to a significant aggravation of myocardial ischemia/reperfusion injury and to an attenuation of the cardioprotective effect of preconditioning (Ferdinandy et al., 2007, 2014; Andreadou et al., 2017). One of the first articles reporting the loss of rapid pacing-induced preconditioning in hypercholesterolemic rabbits was released in Szilvassy et al. (1995). The loss of the infarct size-limiting effect of ischemic preconditioning (Gorbe et al., 2011; Babbar et al., 2013) and late ischemic preconditioning (Yadav et al., 2012) have been shown in different models of diet-induced hyperlipidemia in rats. Detrimental effect of hypercholesterolemia could be due to either increased production and/or decreased removal of highly reactive oxygen and/or nitrogen species (ROS and RNS), such as superoxide, hydrogen peroxide, hydroxyl radicals, and peroxynitrite (Csonka et al., 2016). Diabetes mellitus is a major independent risk factor for acute coronary syndrome (ACS) and causes increased mortality among diabetic individuals (Sethi et al., 2012). Numerous mechanisms have been proposed to contribute to the formation of diabetic cardiomyopathy and myocardial contractile function, including oxidative stress (Singh et al., 2018).

The investigation of mechanisms behind ischemia/reperfusion injury in the presence of hyperlipidemia and other metabolic comorbidities is crucial for testing potential cardioprotective compounds and interventions. Ischemia/reperfusion injury can be modeled with induction of hypoxia/anoxia in a hypoxic chamber, which can be further combined with the application of hypoxic medium. The aforementioned model is widely used in primary cardiac myocyte cultures and cell lines as well (Lecour et al., 2014; Lindsey et al., 2018). We reported previously that simulated ischemia/reperfusion injury causes significant cell death in neonatal rat cardiac myocytes, which can be reversed with an NO-donor treatment (Gorbe et al., 2010). Simulation of hyperlipidemia and hypercholesterolemia *in vitro* is less standardized in the literature. There are only few studies, where lipoprotein or oxidized lipoprotein supplementation was used in cardiac myocyte cultures to induce *in vitro* hyperlipidemia (Cal et al., 2012a, b).

Currently, there is a lack of *in vitro* cell based platforms able to mimic such pathological conditions and to become the gold standard in the development of new effective drug candidates. Therefore, the aim of the present study was to set an *in vitro* medium throughput test system of primary isolated cardiac myocytes, which can be subjected to simulated ischemia/reperfusion and mimics *in vivo* hypercholesterolemia and hyperglycemia. Severity of cell injury and level of oxidative stress could reflect the possible cardioprotective or cardiotoxic effects of tested compounds during preclinical phase of drug development.

## Materials and Methods

These experiments conform to the National Institutes of Health Guide for the Care and Use of Laboratory Animals (NIH Pub. No. 85-23, Revised 1996) and were approved by the local ethics committee at the University of Szeged.

### Study Design

In the present study, we used both primary isolated neonatal and adult rat cardiac myocyte adherent cultures. The following groups were investigated:

(1) normochol (normocholesterolemic control, cell culture medium supplemented with the vehicle of HiChol supplementations)
(2) HiChol 1 (cell culture medium supplemented with hypercholesterolemic medium 1)
(3) HiChol 2 (cell culture medium supplemented with hypercholesterolemic medium 2)
(4) HiChol 3 (cell culture medium supplemented with hypercholesterolemic medium 3).

Each group was tested under the following conditions:

(a) Standard culturing under normoxic condition
(b) Simulated ischemia/reperfusion injury (SI/R)
(c) Simulated ischemia/reperfusion injury + treatment with NO donor drug or its vehicle (a well-known cardioprotective compound) under SI/R
(d) Additional hyperglycemia (high concentration of glucose combined with HiChol supplementation, refers to metabolic disease condition) under normoxic condition
(e) Additional hyperglycemia + simulated ischemia/reperfusion injury.

### Isolation of Neonatal Cardiac Myocytes

Neonatal cardiac myocytes (NRCM) were isolated from new-born (1–3 day old) Wistar rats as described previously (Csont et al., 2010; Bencsik et al., 2014). Briefly, rats were disinfected with 70% ethanol and then euthanized by cervical dislocation. The hearts were rapidly removed and placed in ice cold PBS. Ventricles were separated and minced with fine forceps. Tissue fragments were digested in 0.25% trypsin for 25 min in a conical tube at 37°C. After digestion, the cell suspension was centrifuged (250 × *g* for 15 min at 4°C). Pellet was resuspended in culture medium [Dulbecco’s modified Eagle’s medium (DMEM), supplemented with 10% fetal bovine serum (FBS), L-Glutamine, and Antibiotic/Antimycotic]. This cell suspension was preplated in 6-well plates at 37°C for 90 min to enrich the culture with cardiac myocytes. The non-adherent myocytes were collected and cells were counted and then plated at a density of 10^5^ cells/well in a 24-well plate.

### Isolation of Adult Cardiac Myocytes

Male adult Wistar rats (150 g) were used. Surgery was performed under sodium pentobarbital anesthesia and each animal was heparinised (500 IU/kg) through femoral vein. For cardiac myocyte (ARCM) isolation, hearts were cannulated and perfused retrograde with butanedione monoxide supplemented Krebs–Henseleit solution to wash out the clots and blood. After a 2–4 min solution was changed to collagenase II (8000 U/mL) containing Krebs solution and perfused for 30–45 min. The ventricles were removed and chopped in small pieces and digestion continued for 10 min more. The cell suspension was filtrated and pelleted under gravity, repeated 2–3 times. Under these steps, the Ca^2+^ concentration was increased gradually up to 1 mM. The ratio of the rod shape viable cells was controlled visually under the isolation at each step of the phasic increase of Ca^2+^. We considered isolated adult cardiomyocytes viable when spontaneously contracting and showing rod shape. After cell counting, the cells were plated in laminin-coated wells of a 24-well plate (7500 cell/well) (Markou et al., 2011). To start SI/R experiment minimum 50% viable cells were required by cell counting.

### Tailored Refeed^®^ Supplements

In order to mimic the elevated concentration of cholesterol typical of hypercholesterolemic conditions on cultured primary cardiac myocytes, we identified three increasing cholesterol concentrations suitable for obtaining the desired responses by the cells. However, an *in vivo* hypercholesterolemic condition is usually overlapped by a general hyperlipidemia/dyslipidemia, characterized by a wider array of dysregulated lipids and influenced by multiple factors belonging to genetics, lifestyle and diet. For this reason, we decided to integrate the cholesterol-based supplements with selected lipids, able to generate a more heterogeneous and authentic hypercholesterolemic/hyperlipidemic phenotype in *in vitro* primary cardiac myocytes. The three tailored Refeed^®^ supplements (hiChol1, hiChol2, hiChol3) used in this study were therefore developed by integrating the desired levels of cholesterol with selected adjuvant lipids, in order to strengthen the hypercholesterolemic biological effects and create a more accurate *in vitro* model. Refeed^®^ supplements (Remembrane Srl, Imola, Italy) are a completely defined combination of non-animal derived lipids (NuCheckPrep, Inc., Elysian, MN, United States; Sigma Aldrich, St. Louis, MO, United States; Applichem an ITW, Inc., Chicago, IL, United States) solubilized in 1 mL of ethanol (Sigma Aldrich). 1.5 mL of Refeed^®^ was diluted in 500 mL of complete cell growth medium, the resulting ethanol concentration being less than 1% (vol/vol) in the final medium. The specific tailored Refeed^®^ composition is shown in Table 1. Similar Refeed compositions for different purposes have been previously developed, as described (Poggi et al., 2015; Chatgilialoglu et al., 2017; Cavallini et al., 2018).

**TABLE 1:** Composition of Refeed^®^used for *in vitro* supplementation (hypercholesterolemic medium/hiChol) of cardiac myocytes.

	**HICHOL1**	**HICHOL2**	**HICHOL3**
Cholesterol	1,93	4,83	9,67
Other lipids	2,45	6,14	12,26
**Total lipids**	4.38	10.97	21.93

*Data are the amount (mg) per 500 mL of complete medium.*

### Medium Supplementation and Treatment of Cardiac Myocytes

**FIGURE 1:**
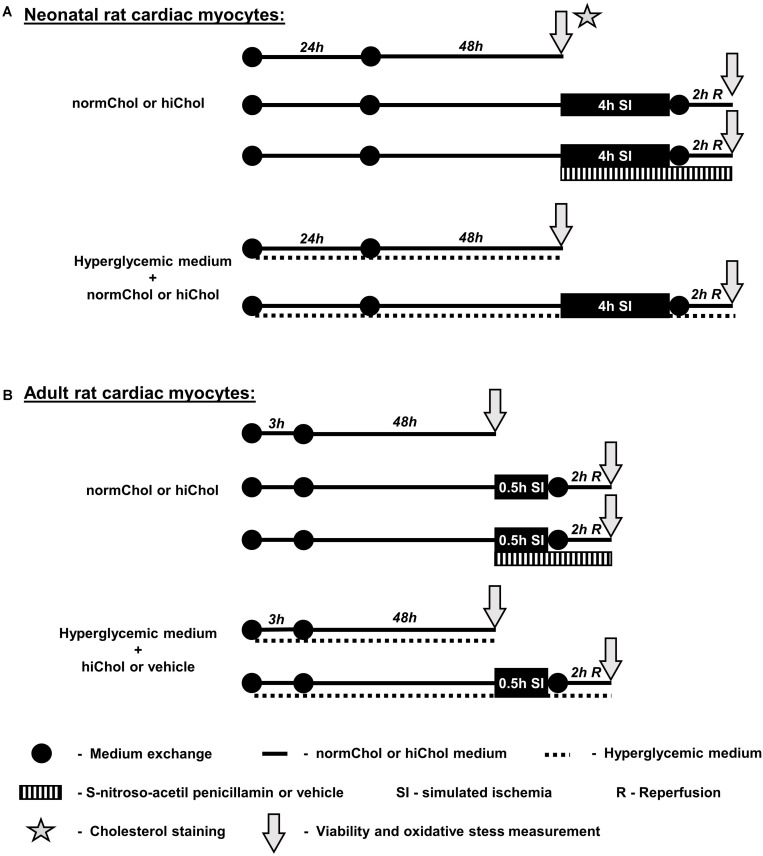
**(A)** Neonatal cardiomyocytes were cultured in normo glycemic or hyperglycemic medium supplemented with vehicle or hypercholesterolemic supplementation (hiChol). Cholesterol staining was to show the effect of hiChol supplementation. Cell viability and oxidative stress, i.e., total reactive oxygen species (ROS) and superoxide level was measured by fluorescent assays after 72 h cultivation. Each group was subjected to normoxia or simulated ischemia/reperfusion injury (SI/R), respectively. Viability and oxidative stress was measured after normoxia or SI/R. **(B)** In adult rat cardiomyocytes treated with vehicle or hiChol supplements cell viability and oxidative stress was measured under normoxia or after SI/R injury.

Neonatal cardiac myocytes were kept at 37°C in a standard CO_2_ incubator (humidified atmosphere of 5% CO_2_) and supplied with growth medium (10% FBS containing DMEM) for 24 h and with proliferation medium (1% FBS) for another 48 h. The adult cardiac myocytes were cultured with same conditions with serum supplemented media for 3 h (5% FBS containing M199) and with growth media (serum free M199) for 48 h (Experimental protocol: Figure 1). Cholesterol supplements (hiChol1, hiChol2, or hiChol3) or vehicle (0.3% ethanol) were added to each series (3 μL into 1 mL culture media) (Figure 1). NO- donor *S*-nitroso-*N*-acetyl penicillamine (10-6 M) was applied during simulated ischemia and reperfusion. High glucose medium contained 4.5 g/L glucose.

### Determination of Cholesterol Content of the Cells by Filipin Staining

To measure the cholesterol content of the cultured cells Filipin staining was used that enables semi-quantification of free cholesterol in biological membranes (Maxfield and Wustner, 2012; Wilhelm et al., 2019). NRCMs were incubated in 300 μL warm D-PBS based Filipin working solution (100 ug/ml) (Sigma, F4767) for 30 min at 37°C. Then we fixed them with 2% paraformaldehyde (10 min at room temperature). After the fixation, cells were permeabilized (digitonin at 500 uM), and then propidium iodide (PI) dye (50 μM, dissolved in D-PBS) was added and incubated for 5 min to assess the cell number. Filipin data were quantified by using a fluorescent microscope (Olympus Fluoview 1000, excitation wavelength: 340 nm; emission wavelength: 410 nm), whereas 20–23 random areas of cell cultures (four different cultures per group) were taken and the integrated density of fluorescence intensity was analyzed by the NIH software ImageJ.

### Simulated Ischemia/Reperfusion (SI/R)

To simulate ischemia/reperfusion injury we used a combination of hypoxic atmosphere (mixture of 95% N_2_ and 5% CO_2_) in a three-gas incubator and a hypoxic solution (in mM: NaCl 119, KCl 5.4, MgSO_4_ 1.3, NaH_2_PO_4_ 1.2, HEPES 5, MgCl_2_ 0.5, CaCl_2_ 0.9, Na-lactate 20, BSA 0.1% pH 6.4, 310 mOsm/L). The culture medium was removed and replaced with the hypoxic solution (without supplementation). Parallel normoxic control was performed, where the culture medium was replaced with normoxic solution (in mM: NaCl 125, KCl 5.4, NaH_2_PO_4_ 1.2, MgCl_2_ 0.5, HEPES 20, MgSO_4_ 1.3, CaCl_2_ 1, glucose 15, taurine 5, creatine-monohydrate 2.5, and BSA 0.1%, pH 7.4, 310 mOsm/L) and the cells kept in the normoxic incubator (Csont et al., 2010; Gorbe et al., 2010; Bencsik et al., 2014; Paloczi et al., 2016). Hypoxic and normoxic solutions were used without modification according to Li et al. (2004). The length of ischemia was 4 h for the neonatal (NRCM) and 30 min for the adult (ARCM) cells. After the ischemic period, the culture medium was replaced and the cells were reoxygenated for 2 h. Cholesterol supplementation was applied again during simulated reperfusion. See for protocol figure (Figure 1). The length of the simulated ischemia is based on our preliminary results and literature. The European Society of Cardiology Working Group Cellular Biology of the Heart has recommended that the combined ischemic and reperfusion times should be selected to result in 50% cell death (Lecour et al., 2014), then cardioprotection can be tested.

### Viability Assays

To assess cell viability, calcein and propidium iodide stainings were performed. Cells were washed with warm D-PBS and calcein solution (1 μM) was added and incubated for 30 min at room temperature in dark chamber. Then the calcein solution was replaced with fresh D-PBS and the fluorescence intensity of each well was detected by fluorescent plate reader (FluoStar Optima, BMG Labtech). Fluorescence intensity was measured in well scanning mode (scan matrix: 10 × 10; scan diameter: 10 mm; bottom optic; no of flashes/scan point: 3; temp: 37°C; excitation wavelength: 490 nm; emission wavelength: 520 nm) (Bencsik et al., 2014).

To express the viability in a ratio of the total cell number we used propidium iodide staining. Propidium iodide (50 μM) and digitonin (500 μM) were added and incubated for 7 min. Then the propidium iodide solution was replaced with warm D-PBS and fluorescence intensity of each well was detected; excitation wavelength: 544 nm; emission wavelength: 620 nm (Bencsik et al., 2014).

### Oxidative Stress Measurements

The presence of general reactive oxygen species (ROS) production was detected with 2,7-dichlorodihydroflourescein diacetate (DCFH-DA) (Sigma; D6883). This fluorogenic dye is widely used to measure general level of oxidative stress, as it measures hydroxyl, peroxyl and other ROS activity within the cell according to manufacturers instruction. The presence of superoxide was detected with an oxidative fluorescent dye dihydroethidium (DHE) (Sigma; D7008). Cardiac myocytes were rinsed with Dulbecco’s Phosphate Buffered Saline (D-PBS), then incubated in 100 μL of 10 μM DHE or DCFH-DA at room temperature for 60 min in a dark chamber. Then the dye solution was replaced with warm D-PBS and fluorescence intensity of each well was detected; excitation wavelength: 530 nm; emission wavelength: 620 nm in case of DHE (Csont et al., 2007) and excitation/emission at 495 nm/529 nm in case of DCFH-DA, as described (Csont et al., 2007; Tao et al., 2007; Kalyanaraman et al., 2012; Ludke et al., 2017).

## Results

### Cholesterol Uptake of Neonatal Rat Cardiac Myocytes

**FIGURE 2:**
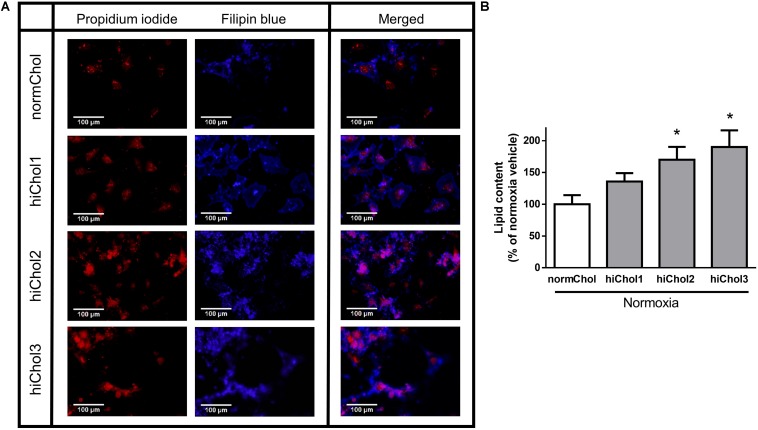
**(A)** Representative images of membrane cholesterol content in neonatal cardiac myocytes after cholesterol supplementation (hiChol1, hiChol2, hiChol3) measured with Filipin staining (for cholesterol content) and propidium iodide staining (for total cell count). **(B)** Membrane cholesterol levels of neonatal cardiac myocytes evaluated (ImageJ software) after cultivation with different supplements (hiChol1, hiChol2, hiChol3). Data are expressed as mean ± SEM compared to normoxia vehicle control group (100%). ^∗^*p* < 0.05 vs. normoxia vehicle (one-way ANOVA, Tukey’s *post hoc*), *n* = 20–23.

Normoxic neonatal cardiac myocytes were treated with cholesterol containing medium with increasing concentrations (hiChol1, hiChol2, hiChol3) of cholesterol to test the uptake by the cells. Filipin staining reflected the cholesterol content of the cardiac myocytes and propidium iodide counterstain reflected the total cell count (representative images Figure 2A). Fluorescence signal analysis showed that cholesterol uptake from the hiChol supplements was efficient and cholesterol content increased in cardiac myocytes at concentration dependent manner (Figure 2B).

### Effect of Hypercholesterolemic Supplementation and Simulated Ischemia/Reperfusion Injury on Neonatal Cardiac Myocytes

**FIGURE 3:**
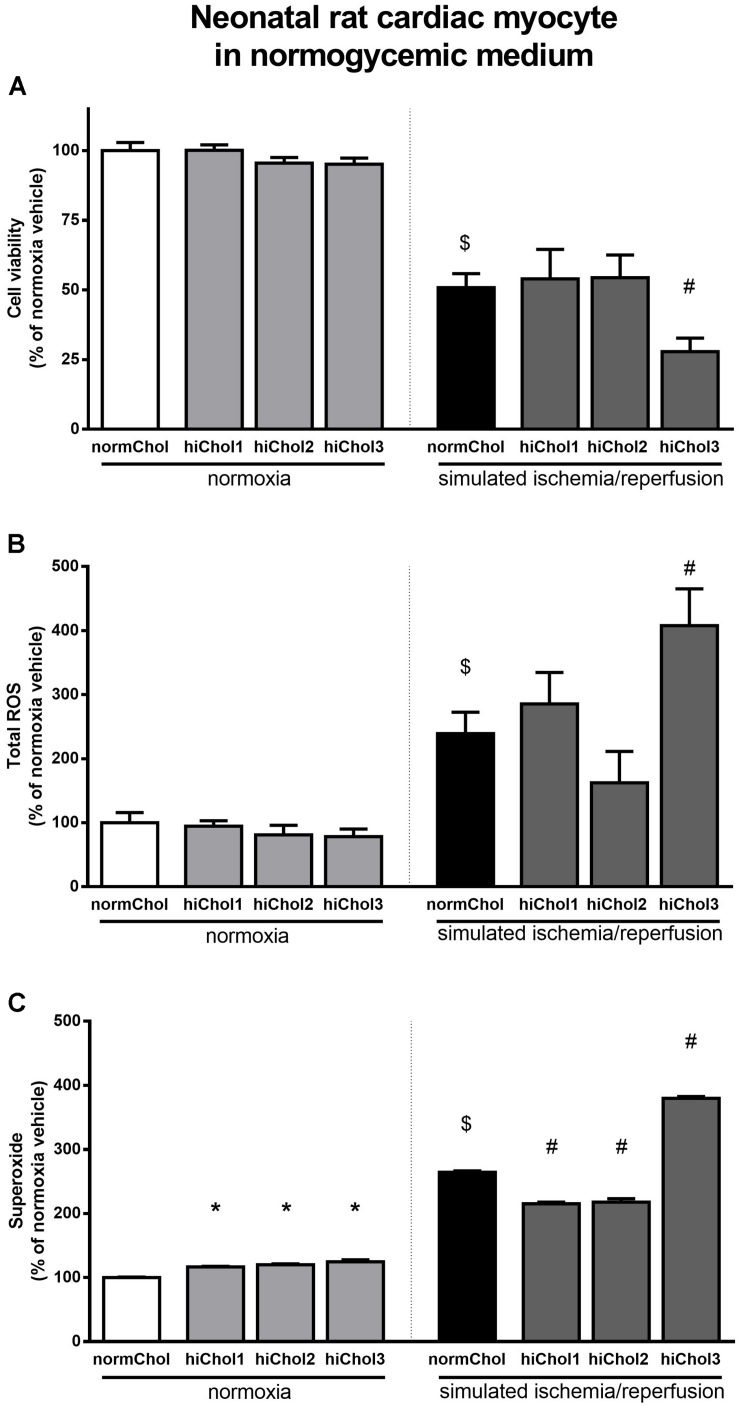
Neonatal rat cardiac myocyte viability was measured **(A)** with calcein AM staining after cultivation with/without hiChol1-3 supplements under normoxic conditions and combined with simulated ischemia/reperfusion injury (SI/R). Total ROS **(B)** and superoxide **(C)** level was measured in neonatal cardiac myocytes treated with cholesterol supplements (hiChol1-3) under normoxia or after SI/R. Data are expressed as mean ± SEM, in comparison to normoxia vehicle control group (100%). $*p* < 0.05 normoxia vehicle vs. SI/R vehicle (*t*-test); ^∗^*p* < 0.05 vs. normoxia vehicle (one-way ANOVA, LSD *post hoc); #p* < 0.05 vs. SI/R vehicle (one-way ANOVA, LSD *post hoc*); *n* = 5–11 “N number denotes the number of wells originated from several technical repeats.”

Under normoxic conditions, the cell viability of neonatal cardiac myocytes was not influenced by the hypercholesterolemic supplementation (Figure 3A). Under normoxic conditions there were no differences in total ROS levels between the groups too (Figure 3B). However, superoxide levels were significantly elevated in all groups (Figure 3C), reflecting some detrimental effect in presence of high level of cholesterol.

Simulated ischemia/reperfusion (SI/R) injury caused significant cell death of normocholesterolemic cardiac myocytes (Supplementary Figure S1A) compared to normoxic groups. Cardiac myocyte viability was significantly decreased with the administration of hiChol3 (Figure 3A). SI/R injury alone increased both total ROS and superoxide levels in normocholesterolemic (normChol) groups, which were further increased in presence of hypecholesterolemic supplementation (hiChol3) (Figures 3B,C).

### Effect of Metabolic Disease Condition and Simulated Ischemia/Reperfusion Injury in Neonatal Cardiac Myocytes

**FIGURE 4:**
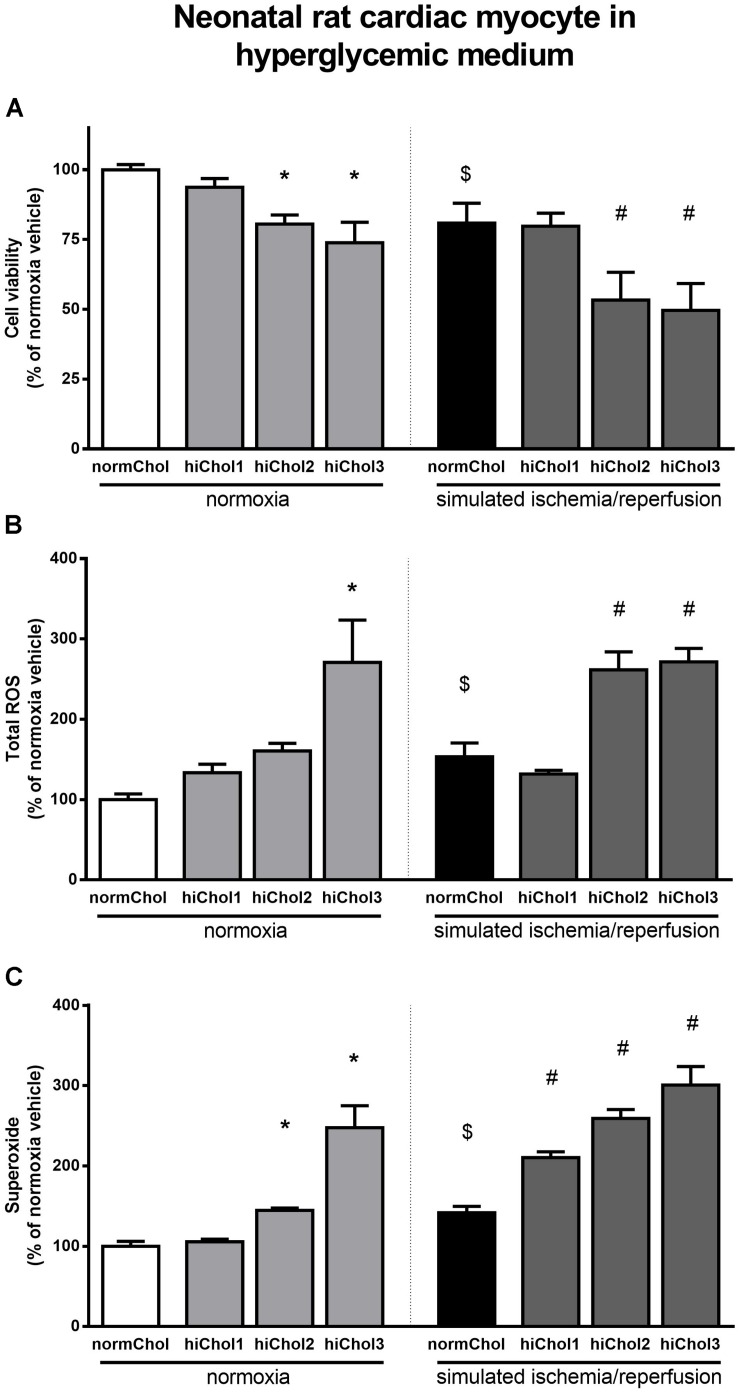
Neonatal rat cardiac myocyte cells cultured in hyperglycemic medium with/without hiChol1-3 supplements. Viability **(A)** was measured with calcein AM staining in normoxia or after SI/R injury. Total ROS **(B)** and superoxide **(C)** level were also measured in normoxia or after SI/R. Data are expressed as mean ± SEM, in comparison to normoxia vehicle control group (100%). $*p* < 0.05 normoxia vehicle vs. SI/R vehicle (*t*-test); ^∗^*p* < 0.05 vs. normoxia vehicle (one-way ANOVA, LSD *post hoc); #p* < 0.05 vs. SI/R vehicle (one-way ANOVA, LSD *post hoc*); *n* = 6–12.

In normoxic condition, when hypercholesterolemic supplementation was applied in combination with high glucose in medium, reduced cell viability was detected at higher concentration of cholesterol (hiChol2 and hiChol3) (Figure 4A). In these groups, total ROS and superoxide levels increased correspondingly (Figures 4B,C). Simulated ischemia-reperfusion further decreased cell viability in hiChol2 and hiChol3, while total ROS and superoxide levels increased (Figure 4).

### Effects of Hypercholesterolemic Supplementation and Simulated Ischemia/Reperfusion Injury in Adult Cardiac Myocytes

**FIGURE 5:**
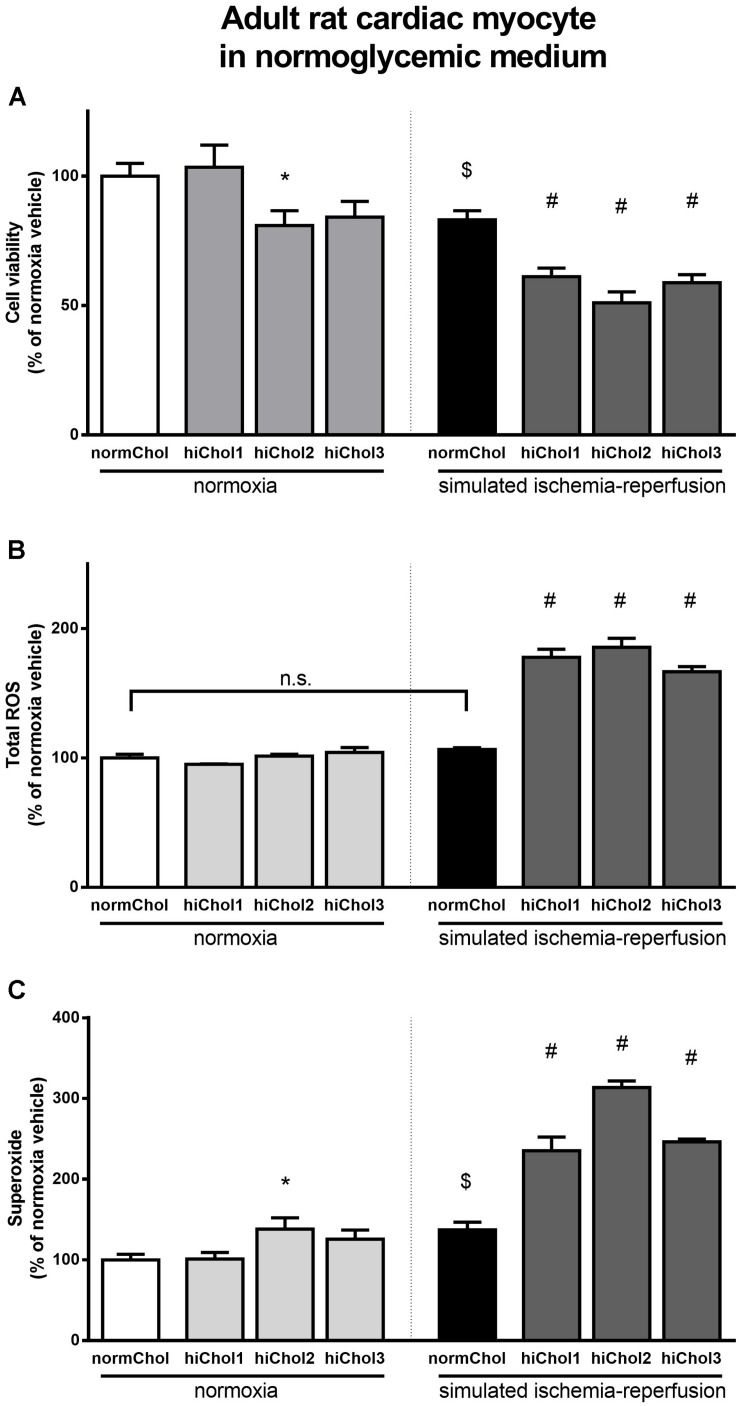
Adult myocardial cell viability **(A)** was measured in normoxia or after simulated ischemia-reperfusion (SI/R) injury with/without hiChol1-3 supplements. Total ROS **(B)** and superoxide **(C)** level was measured too. Total ROS **(B)** and superoxide **(C)** level were also measured in normoxia or after SI/R. Data are expressed as mean ± SEM, in comparison to normoxia vehicle control group (100%). $*p* < 0.05 normoxia vehicle vs. SI/R vehicle (*t*-test); ^∗^*p* < 0.05 vs. normoxia vehicle (one-way ANOVA, LSD *post hoc); #p* < 0.05 vs. SI/R vehicle (one-way ANOVA, LSD *post hoc*); *n* = 5–14 on **(A,C)**; *n* = 3–5 on **(B)**.

We tested the sensitivity of cardiac myocytes isolated from adult rats to hypercholesterolemia. Cell viability was significantly reduced after hiChol2 supplementation of adult cardiac myocytes in normoxia (Figure 5A). The total ROS level was not influenced, but superoxide level was elevated by hiChol2 under normoxic condition (Figures 5B,C).

Simulated ischemia/reperfusion injury caused significant cell death of adult cardiac myocytes (Supplementary Figure S1B). The reduction of cell viability by cholesterol supplementation was further increased when hypercholesterolemia was combined with simulated ischemia/reperfusion injury (Figure 5A). Both total ROS and superoxide showed markedly elevated levels when hypercholesterolemic supplementation and simulated ischemia/reperfusion was combined (Figures 5B,C).

### Effect of Metabolic Disease Condition and Simulated Ischemia/Reperfusion Injury in Adult Cardiac Myocytes

**FIGURE 6:**
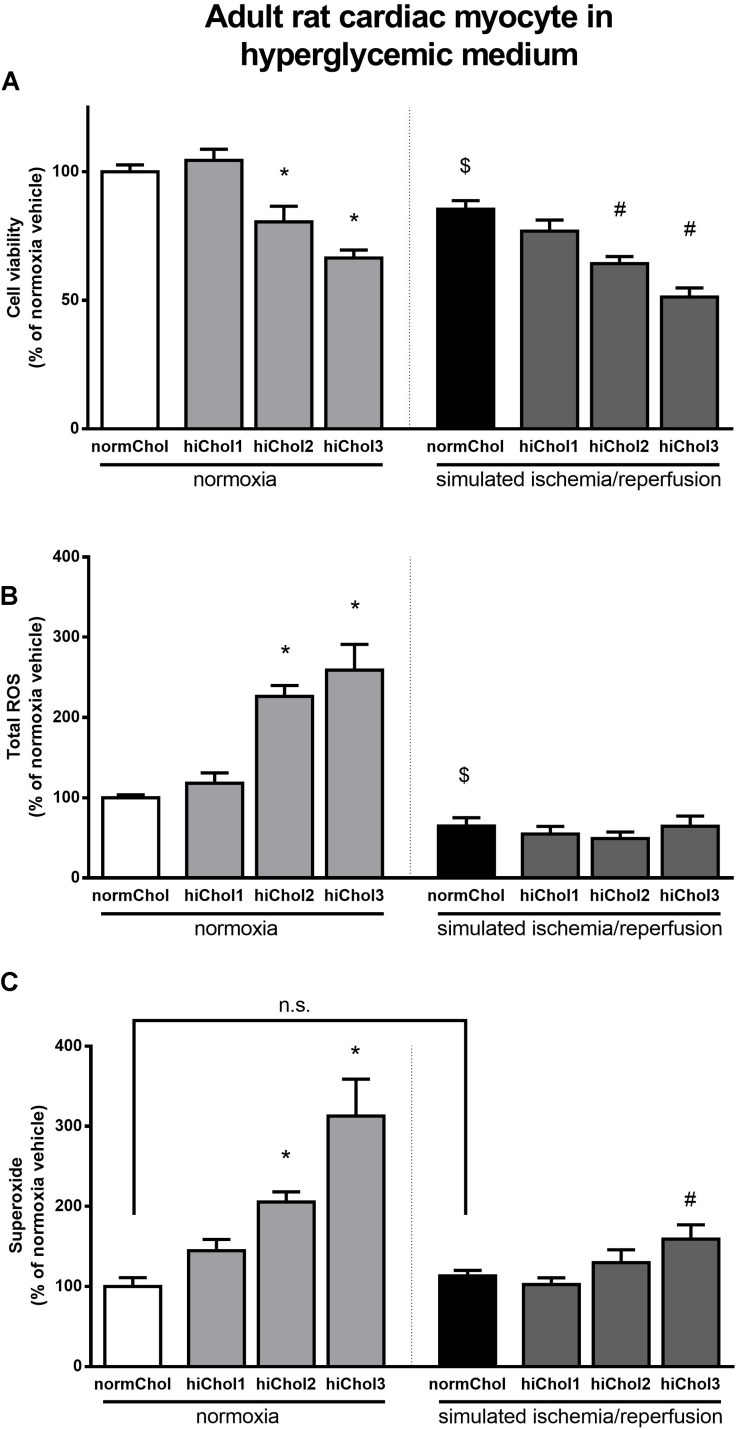
Adult rat cardiac myocyte cells cultured in hyperglycemic medium with/without hiChol supplements. Viability **(A)** was measured with calcein AM staining in normoxia or after SI/R injury. Total ROS **(B)** and superoxide **(C)** level were also measured in normoxia or after SI/R. Data are expressed as mean ± SEM, in comparison to normoxia vehicle control group (100%). $*p* < 0.05 normoxia vehicle vs. SI/R vehicle (*t*-test); ^∗^*p* < 0.05 vs. normoxia vehicle (one-way ANOVA, LSD *post hoc); #p* < 0.05 vs. SI/R vehicle (one-way ANOVA, LSD *post hoc*); *n* = 6–12.

In normoxic condition, when hypercholesterolemic supplementation was applied in combination with high glucose in medium, reduced cell viability was detected at higher concentration of cholesterol (hiChol2 and hiChol3) (Figure 6A). In these groups, total ROS and superoxide levels increased correspondingly in normoxic condition (Figures 6B,C). Simulated ischemia-reperfusion caused similar rate of cell death in hiChol2 and hiChol3 as in normoxic cells, while interestingly total ROS did not changed, superoxide levels increased only in hiChol3 group (Figure 6).

### Cardioprotection Against Simulated Ischemia/Reperfusion Injury in Hypercholesterolemic Neonatal and Adult Cardiac Myocytes

**FIGURE 7:**
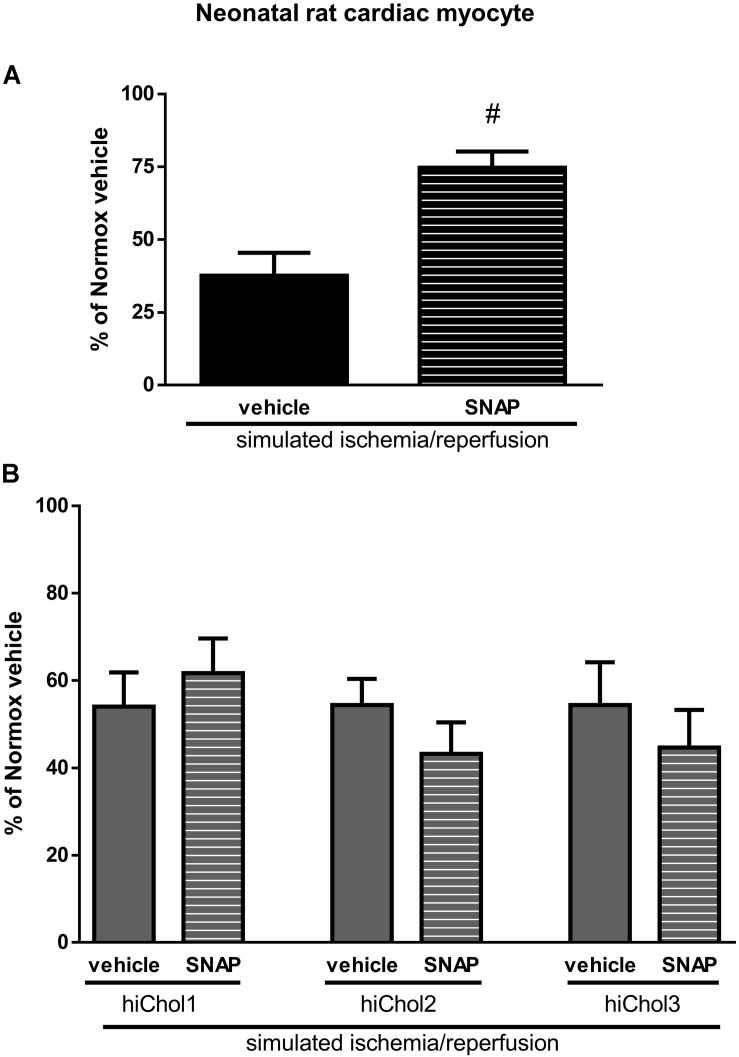
**(A)** SI/R and *S*-niroso-*N*-penicillinamine (SNAP) effect on the cell viability of neonatal cardiomyocytes was detected with calcein-AM. **(B)** Effect of Hypercholesterolemia on protective effect of SNAP against SI/R was tested in each concentration (hiChol1, hiChol2, hiChol3). Data are expressed as mean ± SEM, in comparison to normoxia vehicle control group (100%). ^#^*p* < 0.05 vs. SI/R vehicle (one-way ANOVA, LSD *post hoc*); *n* = 13–15.

**FIGURE 8:**
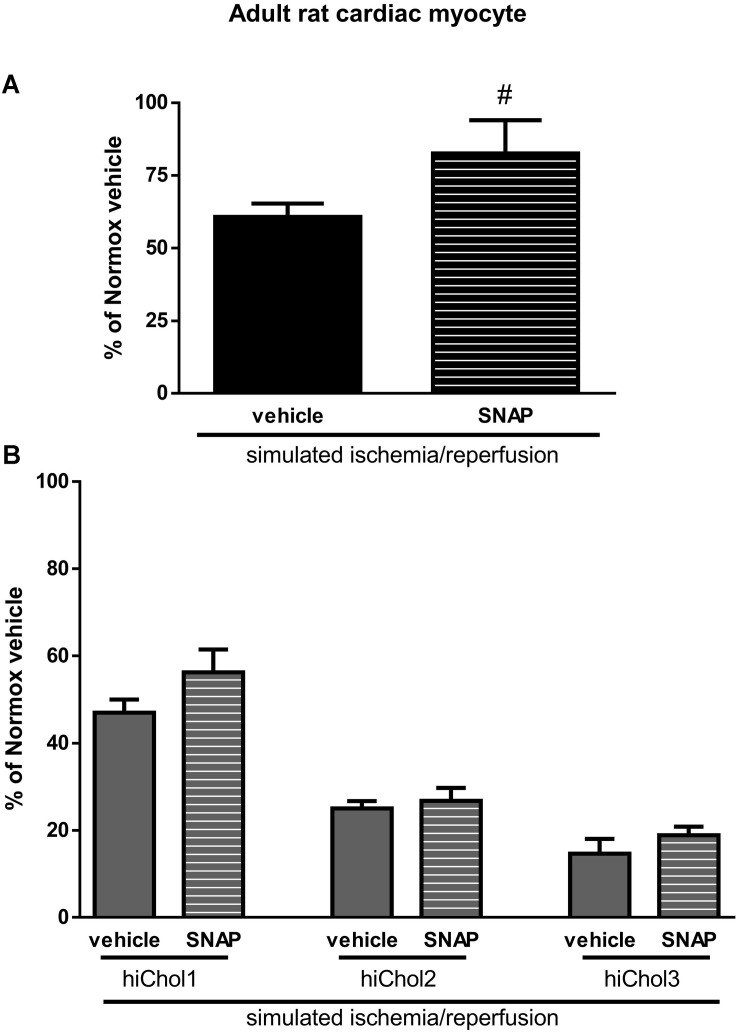
**(A)** SI/R and *S*-niroso-*N*-penicillinamine (SNAP) effect on the cell viability of adult cardiomyocytes was detected with calcein-AM. **(B)** Effect of Hypercholesterolemia on protective effect of SNAP against SI/R was tested in each concentration (hiChol1, hiChol2, hiChol3). Data are expressed as mean ± SEM, in comparison to normoxia vehicle control group (100%). ^#^*p* < 0.05 vs. SI/R vehicle (one-way ANOVA, LSD *post hoc*); *n* = 5–12.

The NO-donor *S*-nitroso-*N*-acetyl penicillamine (SNAP) significantly decreased cell death induced by SI/R injury in neonatal normocholesterolemic cardiac myocytes (Figure 7A). The protective effect of SNAP was abolished in each hiChol supplemented groups (Figure 7B). SNAP significantly decreased rate of cell death induced by SI/R injury in adult normocholesterolemic cardiac myocytes (Figure 8A). Protective effect of SNAP was abolished in each hiChol supplemented groups (Figure 8B).

## Discussion

In the present study, we showan *in vitro* medium throughput cell-based test system of primary isolated cardiac myocytes subjected to simulated ischemia/reperfusion in combination with hypercholesterolemia using tailored hypercholesterolemic supplementation with or without hyperglycemia. HiChol-supplemented rat cardiac myocytes showed reduction of cell viability and increased oxidative stress, which were further aggravated by SI/R and additional hyperglycemia. Moreover, HiChol supplementation blocked the cardiocytoprotective effect the positive control NO-donor SNAP. These results are in accordance to results observed in *in vivo* settings with myocardial infarction and metabolic disease. This is the first demonstration that the combination of the current hypercholesterolemic/metabolic disease medium and SI/R in cardiac myocytes mimics the cardiac pathology of the comorbid heart with I/R and hypercholesterolemia/metabolic disease. This *in vitro* model can be suitable for testing potential drug candidates for cardioprotection.

Hypercholesterolemia is widely accepted as a principal risk factor for CAD (Ferdinandy et al., 2014). Hypercholesterolemia has direct negative effects on the myocardium itself, in addition to the development of atherosclerosis and CAD. In the present study, we observed a concentration-dependent uptake of cholesterol by cardiac myocytes, which formed lipid droplets mainly visible in the cytoplasm. HiChol-supplemented normoxic neonatal rat cardiac myocytes did not show reduced cell viability, but adult rat cardiac myocytes did. Similarly, direct harmful effect of hypercholesterolemia on myocardium has been shown in several experimental animal models. After 10 weeks of cholesterol feeding, both systolic and diastolic impairments were detected without hypertrophy or elevated blood pressure in rabbits (Huang et al., 2004). Reduced myocardial strain was detected with speckle tracking echocardiography in rabbit after 2- and 3-month atherogenic feeding, without atherosclerosis (Liu et al., 2014). It was shown in a hypercholesterolemic rat model that sterol esters affect membrane composition, increase erythrocyte osmotic fragility and decrease antioxidant enzyme levels (Sengupta and Ghosh, 2014). In the present study, the presence of hypercholesterolemia induced an increased level of superoxide formation in both neonatal and adult rat cardiac myocytes in normoxic condition. This finding is in line with *in vivo* data, where increased formation superoxide has been observed in hypercholesterolemic rat myocardium (Onody et al., 2003). Elevated oxidative stress associated with high left ventricular diastolic pressure were observed in *in vivo* and *ex vivo* isolated diet-induced hypercholesterolemic rat hearts as well (Varga et al., 2013). These results shows that the present *in vitro* hypercholesterolemic/metabolic disease cell culture model mimics the *in vivo* settings regarding the deteriorative effects on cardiac myocytes via increased oxidative stress.

As already widely reported in the literature (Lin et al., 2015), lipid dysregulation is often present as a cause or a consequence of many human diseases. Commercially available *in vitro* models do not take into account the influence of lipid dysregulation on most cell properties. Therefore, there is an urgent need for a new generation of *in vitro* models that would be able to mimic pathologies or predisposing conditions also through the consideration of the cell lipidome. Mammalian *in vitro* cells are able to synthesize internally the majority of lipids, lipid building blocks and related precursors they need. However, their preference is to uptake lipids from the cell culture medium, if they are available. Consequently, in the presence of an adequate external source of lipids, most cellular enzymes are down regulated or switched-off. This is why the lipid composition of *in vitro* cells can be modulated by strictly controlling their external supply and a carefully planned feeding strategy grants the possibility to develop efficient *in vitro* models mimicking real *in vivo* conditions (Poggi et al., 2015; Chatgilialoglu et al., 2017). The scope of this work was to develop a hypercholesterolemic comorbidity model of primary cardiac myocytes. In our opinion, the supplementation of increasing concentrations of cholesterol only was a too simplistic way to operate; in fact, *in vivo* hypercholesterolemic conditions are often interconnected with a broader hyperlipidemia/dyslipidemia, characterized by a wider array of dysregulated lipids and influenced by multiple factors belonging to genetics, lifestyle and diet (Castro Cabezas et al., 2018). Frequently, a hypercholesterolemic condition is generated or corroborated by a poor diet quality based on saturated fats and pro-inflammatory lipids (Marais, 2013; Arsenault et al., 2017). For this reason, we decided to integrate the cholesterol-based supplements with selected lipids, thus generating a more heterogeneous and authentic hypercholesterolemic/hyperlipidemic phenotype for our primary cardiac myocyte *in vitro* model. The three tailored Refeed^®^ supplements were therefore developed by integrating the desired levels of cholesterol with selected adjuvant lipids, in order to strengthen the hypercholesterolemic biological effects and create a more accurate *in vitro* model. In our present neonatal rat cardiac myocyte model, hypercholesterolemic supplementation was taken up by cells in a concentration dependent manner and did not influence viability of neonatal cells. Filipin fluorescence intensity showed lipid droplets mainly located in cell cytoplasm. In another study, cardiac myocyte labeled with Filipin shows highest level of cholesterol content in plasma membrane, but also detectable signals can be captured from Golgi apparatus and outer nuclear membrane (Severs, 1982).

There are other, less-controlled external types of lipid supplementation described in the literature in cell culture models, showing direct harmful effect of cholesterol. Cal et al. (2012a, b) describe that the cholesterol uptake from VLDL or LDL lipoprotein levels can affect the regulation of LPR-1 (lipoprotein receptor-related protein 1) receptor expression and the cholesterol accumulation in the ischemic myocardium. Castellano et al. (2011) described the VLDL effect on Ca^2+^ handling and how the hypoxia can further exacerbate this effect. Oxidized forms of lipoproteins can be harmful also directly for the myocardium. Therefore, the present tailored hypercholesterolemic supplementation is suitable for controlled induction of hypercholesterolemia *in vitro.*

In the present study, simulated ischemia/reperfusion was combined with hypercholesterolemic medium. Simulated ischemia/reperfusion induced cell death aggravated harmful effect of hypercholesterolemia in neonatal as well as in adult cardiac myocytes. This finding is in line with majority of *in vivo* animal models of ischemia/reperfusion, in which hypercholesterolemia aggravated the ischemia/reperfusion injury of the myocardium (Andreadou et al., 2017). In the present model, decreased viability of cardiac myocytes was associated with increased levels of total ROS and superoxide anion. One of the most important free radicals generated during hypercholesterolemia is superoxide anion (Landmesser et al., 2000; Napoli and Lerman, 2001). Increased level of ROS and its fundamental role in ischemia/reperfusion injury is an extensively studied phenomenon (Perrelli et al., 2011; Moris et al., 2017; Sinning et al., 2017; Cadenas, 2018; Hernandez-Resendiz et al., 2018). ROS mediated signaling pathway is defined as “redox signaling” (Moris et al., 2017) which was not directly investigated in the present study. ROS modulates several downstream signaling pathways, i.e., the activity of NFkB, which is a well-studied redox-sensitive transcription factor (Frantz et al., 2001). Hypercholesterolemia was the first cardiovascular risk factor to be associated with the loss of cardioprotection due to deterioration of several signaling mechanisms (Ferdinandy et al., 2007, 2014), including disruption of NO-cGMP-PKG pathway (Giricz et al., 2009), KATP signaling (Csonka et al., 2014), Connexin43 distribution (Gorbe et al., 2011), inhibition of opening of mitochondrial permeability transition pores (Yadav et al., 2010), among several other (Andreadou et al., 2017).

To further validate our system, we used a well-known cardioprotective NO-donor to test if its cardiocytoprotective effect is also blocked by hyperchoelsteolemia in our *in vitro* system. Here we have found that the NO-donor SNAP protected both neonatal and adult normocholesterolemic cardiac myocytes against SI/R injury, but not the hypercholesterolemic cardiac myocytes. These results further validated our present *in vitro* I/R and hypercholesterolemic model is suitable for testing cardioprotective in the presence of hypercholesterolemic comorbidity.

Ischemic heart disease associates with several risk factors and comorbidities, like aging and diabetes. Several studies investigated the effect of hyperglycemia on ischemic heart and cardioprotection in different experimental animal models of diabetes and in diabetic patients. Studies showed that the presence of diabetes might interfere with the cardioprotective mechanisms, attenuating the effectiveness of these therapeutic strategies (Ferdinandy et al., 2014). Therefore, here we investigated the presence of hyperglycemia in addition to hypercholesterolemia in isolated primary cardiac myocytes. Here we have found that the combination of hypercholesterolemia and hyperglycemia mimicking metabolic disease worsened the survival of cardiac myocytes even in normoxic condition. Reduction in cell viability and increase in the level of oxidative stress were further aggravated in ischemic neonatal cardiac myocytes. In case of adult cardiac myocytes, SI/R injury interestingly total ROS did not changed, and superoxide levels increased only in hiChol3 group. We have previously found that acute hyperglycemia *in vivo* did not influence infarct size in rat acute myocardial model, but abolished cardioprotective effect of remote ischemic preconditioning (Baranyai et al., 2015). In a diabetic mice model, the exacerbation of heart failure after MI has been observed via increasing NAD(P)H oxidase-derived superoxide. These results further prove the validity of our present *in vitro* I/R and hypercholesterolemic/metabolic disease model is suitable for testing cardioprotective compounds in the presence of hypercholesterolemic/metabolic disease comorbidity (Matsushima et al., 2009).

### Limitations

The mechanisms of increased oxidative stress, i.e., ROS producing enzymes and/or decreased antioxidant capacities were out of the scope of the present study.

## Conclusion

This is the first comorbidity cell-based *in vitro* test system of ischemia/reperfusion injury and hypercholesterolemia/metabolic diseasemimics the *in vivo* comorbidity condition of myocardial ischemia/reperfusion injury. The present test system should be considered as a screening platform for testing potential cardiocytoprotective drug candidates in the presence of these comorbidities.

## Data Availability Statement

All datasets generated for this study are included in the article/Supplementary Material.

## Ethics Statement

The animal study was reviewed and approved by the local ethics committee at the University of Szeged Animal Ethics Committee (I-74-52/2012 MAB) and at Semmelweis University, Budapest, Hungary, and by the National Scientific Ethical Committee on Animal Experimentation and permitted by the government (Food Chain Safety and Animal Health Directorate of the Government Office for Pest County) (PE/EA/1784-7/2017).

## Author Contributions

AM performed the data analysis, prepared all figures, and wrote the manuscript. ÁS performed simulated ischemia/reperfusion testing. JPá performed Calcein and PI viability assays. JPi performed Filipin staining and image collection and data analysis. BK performed simulated ischemia/reperfusion testing and viability assays. PP developed hypercholesterolemic supplementation. PF performed project planning, and wrote the manuscript. AC developed hypercholesterolemic supplementation, prepared table, and wrote the manuscript. AG performed project planning, performed data analysis, and wrote the manuscript.

## Conflict of Interest

AC and PP are co-founders and shareholders of Remembrane S.r.L., a manufacturer of lipid supplements for use in *in vitro* culturing. PF is an owner and CEO of Pharmahungary Group, a group of R&D companies. The remaining authors declare that the research was conducted in the absence of any commercial or financial relationships that could be construed as a potential conflict of interest.

## Supplementary Material

FIGURE S1 Simulated ischemia/reperfusion (SI/R) injury caused significant cell death of cardiac myocytes, both in neonatal (NRCM) **(A)** and adult (ARCM) **(B)** cardiomyocytes. Vehicle alone did not cause further damage in cell viability beyond SI/R effect. ^∗^*p* < 0.05 vs. Normoxia (one-way ANOVA, LSD *post hoc*); *n* = 5–11.
