# Supplementary figures and images for: A Comorbidity Model of Myocardial Ischemia/Reperfusion Injury and Hypercholesterolemia in Rat Cardiac Myocyte Cultures
Source: Front Physiol. 2020 Jan 9;10:1564. doi: 10.3389/fphys.2019.01564 (PMC6962358; doi:10.3389/fphys.2019.01564)

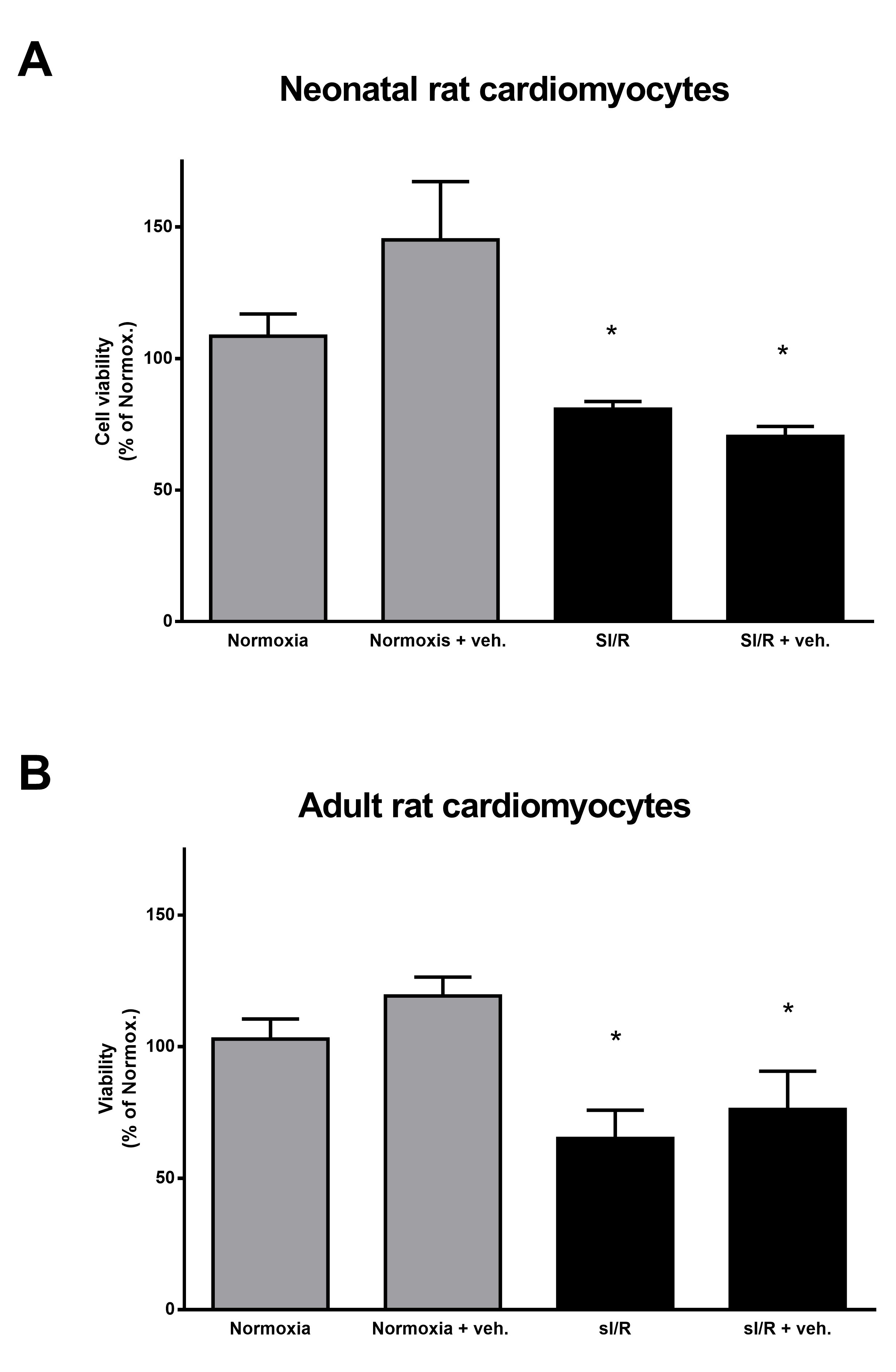

Supplement: FIGURE S1 — Simulated ischemia/reperfusion (SI/R) injury caused significant cell death of cardiac myocytes, both in neonatal (NRCM) (A) and adult (ARCM) (B) cardiomyocytes. Vehicle alone did not cause further damage in cell viability beyond SI/R effect. ∗p < 0.05 vs. Normoxia (one-way ANOVA, LSD post hoc); n = 5–11. [file Image_1.JPEG]
